# Common Evolutionary Origin for the Rotor Domain of Rotary Atpases and Flagellar Protein Export Apparatus

**DOI:** 10.1371/journal.pone.0064695

**Published:** 2013-05-28

**Authors:** Jun-ichi Kishikawa, Tatsuya Ibuki, Shuichi Nakamura, Astuko Nakanishi, Tohru Minamino, Tomoko Miyata, Keiichi Namba, Hiroki Konno, Hiroshi Ueno, Katsumi Imada, Ken Yokoyama

**Affiliations:** 1 Department of Molecular Biosciences, Kyoto Sangyo University, Motoyama Kamigamo, Kita-ku, Kyoto, Japan; 2 Graduate School of Frontier Biosciences, Osaka University, Osaka, Japan; 3 Department of Applied Physics, Graduate School of Engineering, Tohoku University, Sendai, Miyagi, Japan; 4 Precursory Research for Embryonic Science and Technology, Japan Science and Technology Agency, Kawaguchi, Saitama, Japan; 5 Riken Quantitative Biology Center, Osaka, Japan; 6 Imaging Research Division, Bio-AFM Frontier Research Center, Kanazawa University, Kanazawa, Ishikawa, Japan; 7 Department of Physics, Faculty of Science and Engineering, Chuo University, Bunkyo-ku, Tokyo, Japan; 8 Graduate School of Science, Osaka University, Toyonaka, Osaka, Japan; University of Cambridge, United Kingdom

## Abstract

The V_1_- and F_1_- rotary ATPases contain a rotor that rotates against a catalytic A_3_B_3_ or α_3_β_3_ stator. The rotor F_1_-γ or V_1_-DF is composed of both anti-parallel coiled coil and globular-loop parts. The bacterial flagellar type III export apparatus contains a V_1_/F_1_-like ATPase ring structure composed of FliI_6_ homo-hexamer and FliJ which adopts an anti-parallel coiled coil structure without the globular-loop part. Here we report that FliJ of *Salmonella enterica* serovar Typhimurium shows a rotor like function in *Thermus thermophilus* A_3_B_3_ based on both biochemical and structural analysis. Single molecular analysis indicates that an anti-parallel coiled-coil structure protein (FliJ structure protein) functions as a rotor in A_3_B_3_. A rotary ATPase possessing an F_1_-γ-like protein generated by fusion of the D and F subunits of V_1_ rotates, suggesting F_1_-γ could be the result of a fusion of the genes encoding two separate rotor subunits. Together with sequence comparison among the globular part proteins, the data strongly suggest that the rotor domains of the rotary ATPases and the flagellar export apparatus share a common evolutionary origin.

## Introduction

**Figure 1:**
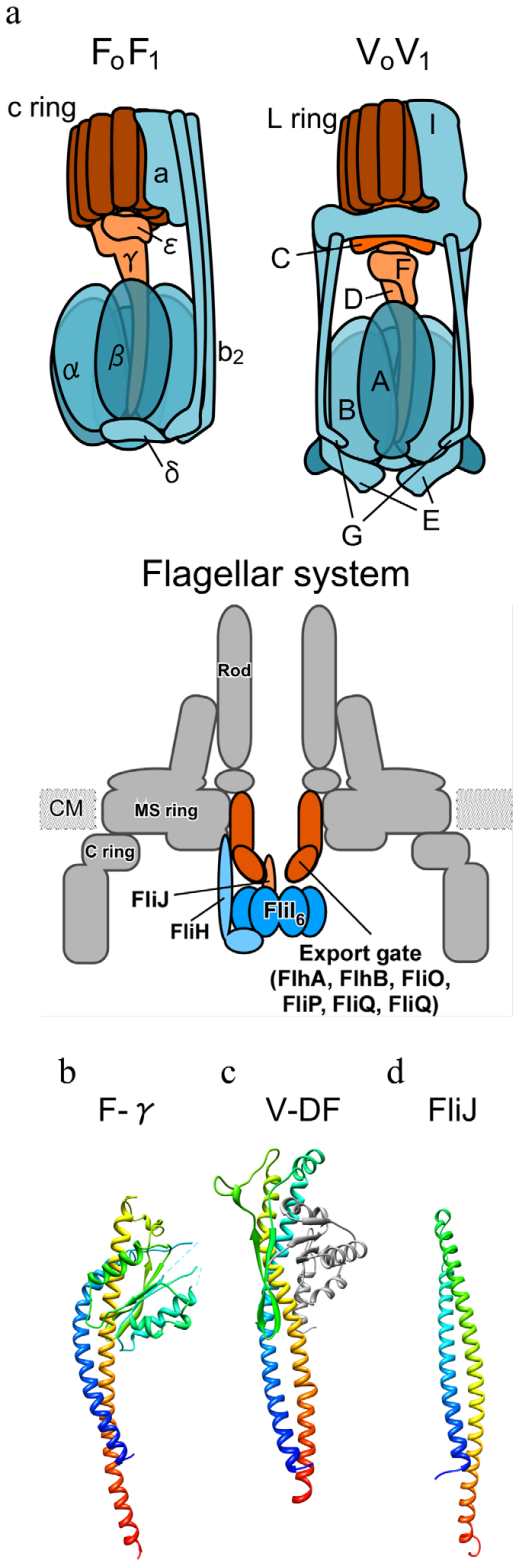
Structure of rotary ATPases and rotors. (**a**) Schematic model of prokaryotic F_o_F_1_ (left) and V_o_V_1_ (right). Rotor subunits (D, F, V_o_-d in V_o_V_1_, γ and ε in F_o_F_1_) are presented in brown. Rotor rings composed of hydrophobic c subunit are presented in dark brown. Peripheral stators (EG and V_o_-a in V_o_V_1_, b_2_, δ, and F_o_-a in F_o_F_1_) are presented in sky blue. Schematic model of the flagellar type III export apparatus (lower). The export apparatus consists of a proton-driven export gate made of six integral membrane proteins, FlhA, FlhB, FliO, FliP, FliQ and FliR, and a water-soluble ATPase complex composed of FliH, FliI, and FliJ. The FliI_6_-FliJ shows remarkable structural similarity to F_1_ and V_1_. The amino acid sequence of FliH shows sequence similarity to peripheral stalk E subunit of V_o_V_1_. (**b**) Crystal structure of γ subunit from bovine F_o_F_1_ (PDB: 1E79). (**c**) Crystal structure of DF subunit of *E.hirae* V_o_V_1_ (PDB: 3AON). (**d**) Crystal structure of FliJ of *Salmonella* ***t*** *yphimurium* (PDB:3AJW).

Two types of rotary ATPases are found in biological membranes; V_o_V_1_(V type ATPase) and F_o_F_1_ (F type ATPase) [1]–[4]. Evolutionary counterparts of the eukaryotic V_o_V_1_ are found in most archea and some bacteria (often referred to as A-type ATPase). Both V_o_V_1_ and F_o_F_1_ couple ATP synthesis and hydrolysis to proton translocation across the membrane by rotation of the rotor apparatus against the surrounding stator which includes the catalytic A_3_B_3_ or α_3_β_3_ hexamer (Fig. 1a). Sequence and structural comparison of the two ATPases indicate significant homology between the catalytic subunits, but not between the subunits of the central rotor domain [5]. For example, V_o_V_1_ lacks a counterpart of the rotor shaft F_1_-γ subunit [1], [4], [5].

The minimal ATP-driven rotary unit of F_o_F_1_ is F_1_, which is comprised of three different proteins with a stoichiometry of α_3_β_3_γ. The F_1_-γ contains two distinct domains; a coiled-coil domain that penetrates the central cavity of the α_3_β_3_ cylinder from the top to the bottom, and a globular domain containing a α/β fold which makes contact with the F_1_-ε (Figs. 1a,b).

For V_o_V_1_, the minimal rotary unit consists of the A_3_B_3_D subunits [6]. In addition, analysis of the secondary structure of the D subunit predicts the presence of long α-helices at both the amino and carboxyl termini as found in the F_1_-γ (Fig. 1b, Supplementary Fig. S1a). On the basis of this, it has been suggested the D subunit is likely to be a structural and mechanistic analog of F_1_-γ despite the lack of any significant sequence similarity [5]. However, F_o_F_1_ lacks a counterpart of the V_1_-F subunit [1], [4], [5], which has a typical globular α/β fold [7] (Fig. 1c). Intriguingly, the X-ray structure of the entire V_1_ of *T. thermophilus* revealed that the central rotor subunit contains both the coiled-coil (D) and globular domains (F), suggesting that the V_1_-F and V_1_-D subunits together form the counterpart of the α/β domain of F_1_-γ [8].

Mulkidjanian *et al.* have proposed a scenario to explain the origin of rotary ATPases, where rotary ATPases share an evolutionary origin with the bacterial flagellar and non-flagellar type III export systems [5]. The flagellar export apparatus consists of a membrane-embedded export gate composed of FlhA, FlhB, FliO, FliP, FliQ and FliR, and a water-soluble ATPase complex consisting of FliH, FliI, and FliJ (Fig. 1a, lower panel) [9]. Components of the flagellar ATPase complex, which allows the export gate to efficiently utilize proton motive force across the cytoplasmic membrane as an energy souse for protein translocation [10], [11], exhibit extensive structural and sequence similarity to catalytic and rotor subunits of rotary ATPases. For instance, the atomic structure of FliI ATPase of the flagellar export apparatus is remarkably similar to the F_1_ β/α and V_1_ A/B subunits [12]. FliJ, which is a soluble export component protein, also shows a striking structural similarity to the coiled-coil region of F_1_-γ [13]. This structural motif is also seen in the YscO-like protein, CT670 from *Chlamydia tracomatis*, a FliJ homolog of the non-flagellar type III export apparatus [14]. Interestingly, FliJ promotes the formation of a homo-hexamer of FliI by binding to the center of the ring, facilitating the FliI ATPase activity [9]. A very similar arrangement is observed in the F_o_F_1_-ATPase with the antiparallel α-helical coiled coil formed by the N- and C-terminal regions of the γ subunit penetrating into the central cavity of the α_3_β_3_ ring. These findings indicate that the type III export system has a F_1_- or V_1_-like structure and share a common evolutionary origin.

Here we aimed to explore the evolutionary relationship between the rotor domains of the V_o_V_1_ and F_o_F_1_ and the type III export system. FliJ formed chimeric complex with A_3_B_3_ of V_o_V_1_ and promoted their ATPase activity. Single molecular analysis indicates anti-parallel coiled coil structure (FliJ structure) protein functions as a rotor axis. These results strongly suggest that the FliJ structure proteins are the ancestral subunit of the rotor subunit of rotary ATPases. We also discuss the evolutionary relationship between globular domain of F_1_-γ, and V_1_-F.

## Methods

### Proteins

The A_3_B_3_JL and A_3_B_3_DF_f_ expression plasmids were constructed from His-tagged V_1_ (A_(His-10/C28S/S232A/T235S/C255A/C508A)3_B_(C264S)3_D_(E48C/Q55C)_F) as described in Figs. S3 *a*, S4*a*. The A_3_B_3_JL (A_(His-10/C28S/S232A/T235S/C255A/C508A)3_B_(C264S)3_JL), A_3_B_3_DF_f_, and A_3_B_3_ were expressed in *E. coli* and the expressed enzymes purified by Ni^2+^-affinity chromatography (Qiagen) followed by ion exchange on a RESOURCE Q column (GE healthcare) [6]. The purified His-tagged enzymes were biotinylated at two cysteines using 6-[*N*-[2-(*N*-maleimide)ethyl]-*N*-piperazinylamide]hexyl-D-biotinamide (Dojindo, Kumamoto, Japan). The bound ADP in each enzyme was partially removed by successive EDTA-heat treatment [18].

FliJ was expressed in BL21(DE3)pLysS and purified as described previously [13]. FliJ(C32T/I61C/I67C) was created by QuikChange mutagenesis (Stratagene), expressed in BL21(DE3)pLysS, biotinylated and purified as the same protocol used for FliJ(C32T/I67C) [13]. A_3_B_3_ was expressed and purified as previously described [15]. The A_3_B_3_-FliJ complex was reconstructed from the purified FliJ and A_3_B_3_. FliJ and A_3_B_3_ were mixed and incubated overnight at 23°C. The mixture was applied to a HilLoad Superdex 200 column in 20 mM MOPS-NaOH pH 7.0 and 100 mM NaCl. The fraction containing the A_3_B_3_-FliJ complex was pooled and stored for future use.

### Rotation experiments

Biotinylated FliJ(C32T/I61C/I67C) and His-tagged A_3_B_3_ were mixed and incubated overnight at 23°C. The A_3_B_3_-FliJ mixture was applied to 5-ml polypropylene column containing Ni-NTA agarose (Qiagen), and A_3_B_3_-FliJ complex was eluted with a sodium phosphate buffer (pH 7.2) containing 200 mM imidazole and 300 mM NaCl. BSA buffer, which is buffer A (50 mM Tris-Cl, pH 8.0, 100 mM KCl, 2 mM MgCl_2_) containing 2 mg ml^−1^ BSA, was infused into the flow cell to prevent nonspecific binding, and then the A_3_B_3_-FliJ complexes diluted 1∶10 into BSA buffer were attached on the Ni-NTA glass and incubated for 15 minutes at 23°C. Unbound molecules were washed out with BSA buffer, and streptavidin-coated gold spheres with a diameter of 40-nm (BioAssay Works) diluted into BSA buffer and infused into the flow cell. After incubation for 15 minutes at 23°C, unbound gold spheres were washed out with buffer A. After infusion of buffer A containing 4 mM Mg-ATP, 2.5 mM phosphoenol pyruvate, and 0.5 mg ml^−1^ pyruvate kinase, rotation of the gold spheres were observed by a dark-field microscope (BX53, U-DCW, UPlanFLN 100×, PE 5×; Olympus) and recorded with a high-speed camera (ICL-B0620M-KC, IMPERX) at 0.8–1.6-ms intervals at 23°C. For the rotation assay of A_3_B_3_DF_f_ or A_3_B_3_JL, the biotinylated enzyme (1–5 nM) in buffer A was applied to the flow cell and incubated for a few minutes at 23°C. Streptavidin-coated magnetic beads (100–300 nm) and Ni^2+^-NTA coated cover glasses were prepared as previously described [20], [21]. Unbound enzyme was washed out with 20 µl of buffer A. Then, 20 µl of buffer A with 2 mg ml^−1^ BSA was infused to the flow cell and incubated for <30 s to prevent nonspecific binding. The BSA solution in the chamber was washed out with 20 µl of buffer A. Then, buffer A containing streptavidin coated magnetic beads (10^10^<10^11^ particles ml^−1^) were infused into the flow cell and incubated for a few minutes. Unbound beads were washed out with 20 µl of buffer A. After infusion of 80 µl of buffer A containing Mg-ATP at the indicated concentration, 2 mM MgCl_2_, 2.5 mM phosphoenol pyruvate, and 0.5 mg ml^−1^ pyruvate kinase, rotation of the bead was recorded with a high speed camera (Eclips, IN) at 1000 frames per second (f.p.s.) using a phase-contrast microscope (IX70, Olympus) with ×100 objective lens (N.A., 1.30, Olympus) at 23°C. Images were captured as an 8-bit AVI file. The centroid of the bead images was calculated [20], [21].

### Electron cryo-microscopy and image analysis

Sample grids were prepared by applying 3 µl of a protein solution containing the *in vitro* reconstructed A_3_B_3_J rings (150 µg/ml) onto a holey carbon grid (Quantifoil R0.6/1.3, Quantifoil Micro Tools, Jena, Germany), which had been glow discharge for 20 s before use. The grids were blotted onto filter paper for 5 s to remove excess solution, vitrified in liquid ethane at −196°C using a Vitrobot (FEI, Eindhoven, Netherlands) and transferred into a liquid nitrogen storage capsule. Particle images were recorded with a 4 K×4 K Slow Scan CCD camera (TemCam-F415MP, TVIPS) mounted on a JEM-3200FSC electron microscope (JEOL, Tokyo, Japan), equipped with a liquid-helium cooled specimen stage, a Ω-type energy filter and a field-emission electron gun operated at an accelerating voltage of 200 kV. Electron micrographs were collected at 50 K with a magnification of ×140,000, corresponding to 1.07 Å/pixel. Focal pairs of the micrographs were recorded at a defocus between 1.5 and 2.5 µm for the first micrograph and at a defocus between 3.5 and 5.5 µm for the second. Electron dose was set to 30 e^−^/Å^2^ for both micrographs. The particle images were processed using the EMAN software package [22]. The focal pairs were merged using FOCALPAIR [22], [23]. The defocus amplitude, envelope and noise values were determined using CTFIT [22]. Micrographs showing significant astigmatism or drift were discarded. The particle images were selected with BOXER [22] and the boxed particle images were aligned and classified using REFINE2D.PY [22].

### Phylogenetic tree analysis

All sequences used in this study were aligned using the MAFFT program (http://mafft.cbrc.jp/alignment/server/) [24]. Phylogenetic and molecular evolutionary analyses were conducted using MEGA version 5 [25]. The phylogenetic tree was constructed using the Maximum-Likelihood (ML) method under the Jones-Taylor-Thornton model by using MEGA.

## Results

### Reconstitution of A_3_B_3_J

**Figure 2:**
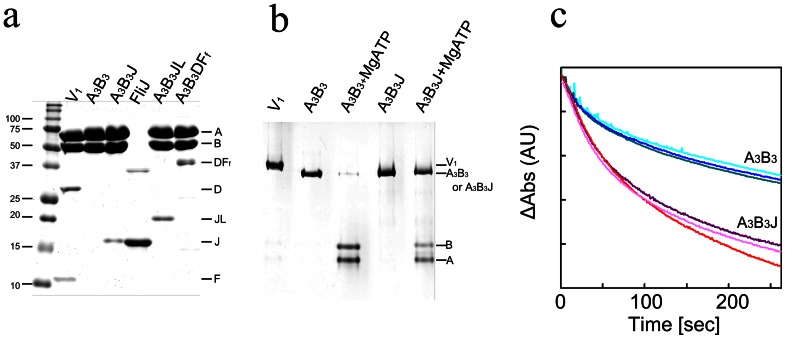
Analysis of A_3_B_3_J. (**a**) SDS-PAGE analysis of A_3_B_3_J and A_3_B_3_DF_f_. (**b**) Analysis of disassembly of A_3_B_3_ and A_3_B_3_J on Native PAGE. Each complex was incubated without nucleotide, or with 1 mM MgATP at 37°C for 1 h, followed by separation by Native PAGE. (**c**) ATP hydrolysis activity of A_3_B_3_ and A_3_B_3_J. Time courses of ATP hydrolysis catalyzed by A_3_B_3_ (blue lines) and A_3_B_3_J (red lines) at 25°C and at 4 mM MgATP. The reaction was started by the addition of 20 µl of 1 µM enzyme solution to 2 ml of assay mixture.

Structural sequence alignment has previously revealed that FliJ shares some amino acid conservation with F_1_-γ [13]. The structural and sequence similarity suggests that FliJ may behave as a counterpart of rotor subunit of rotary ATPases. To test this possibility, we investigated whether FliJ binds to the center of the A_3_B_3_ ring of *T. thermophilus*, forming the A_3_B_3_J complex. A_3_B_3_ was mixed with excess amounts of FliJ, and the mixture incubated overnight at room temperature. The mixture was applied to a gel filtration column to remove free FliJ. The retention time of the complex peak was almost identical to that of A_3_B_3_ alone (Fig. S2a). SDS-PAGE analysis revealed that FliJ co-eluted with A_3_B_3_, indicating the formation of the A_3_B_3_J complex (Fig. 2a). Previous studies have shown that V_1_ and A_3_B_3_D are resistant to ATP-induced dissociation [15] while A_3_B_3_ completely dissociates into monomeric A and B subunits by incubation with 1 mM ATP for 30 min (Fig. 2b). In contrast, the A_3_B_3_J complex is at least partially resistant to ATP-induced dissociation (Fig. 2b), indicating that the presence of FliJ stabilizes the A_3_B_3_ complex to a significant degree. ATP hydrolysis by V_1_ or A_3_B_3_D proceeds at a steady rate for a few minutes, then decelerates slowly, due to ADP inhibition [16]. In contrast, the ATPase activity of A_3_B_3_ decelerates rapidly, reaching a steady state rate within a few minutes owing to rapid ATP-induced dissociation of A_3_B_3_ [16]. In the case of A_3_B_3_J, the ATP hydrolysis profile is similar to that of V_1_ or A_3_B_3_D exhibiting continuous ATP hydrolysis activity after an initial burst phase (Fig. 2c). The turnover rate of A_3_B_3_J is ∼7.0 s^−1^, almost twice that of A_3_B_3_ (∼4.0 s^−1^). Together, these results suggest that FliJ stabilizes the A_3_B_3_ hexamer by binding to A_3_B_3_ and promotes continuous ATPase activity in a similar manner to the V_1_-D subunit.

### Electron cryo-microscopy analysis of A_3_B_3_J

**Figure 3:**
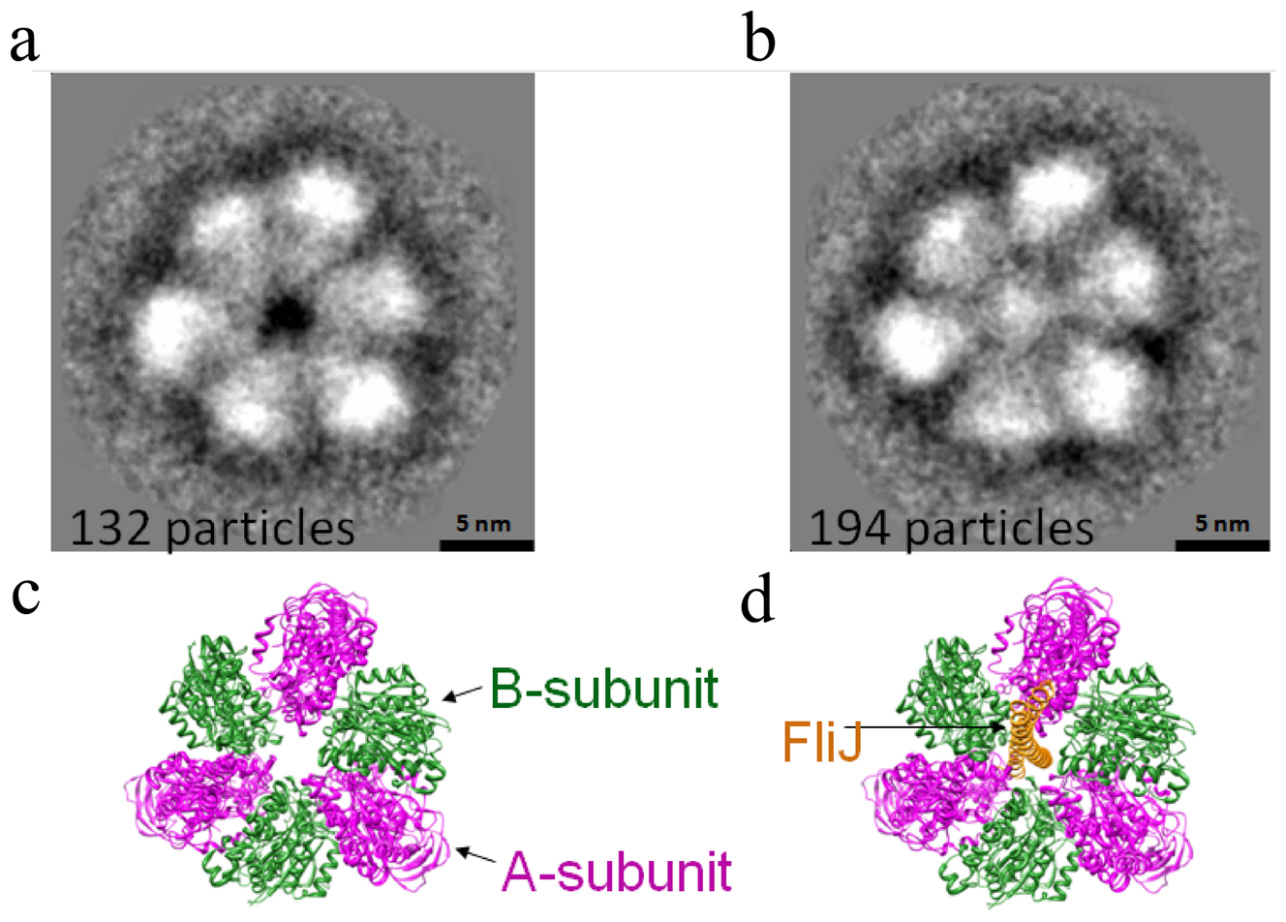
Structure of A_3_B_3_ and A_3_B_3_J. Averaged cryo-EM image of A_3_B_3_ (**a**) and A_3_B_3_J (**b**) are shown. Possible ribbon models of A_3_B_3_ (**c**) and A_3_B_3_J (**d**) are also indicated below each EM image.

In order to identify the location of FliJ in the A_3_B_3_J complex, we analyzed the images of frozen-hydrated A_3_B_3_J and A_3_B_3_ particles embedded in vitreous ice by electron cryo-microscopy. Since the distribution of the particle orientation was strongly biased to end-on view, we aligned and averaged the end-on images of each particle. The averaged image of A_3_B_3_ shows a hetero-hexameric ring structure with an unoccupied central hole of 2 nm in diameter (Fig. 3a), consistent with the crystal structure of A_3_B_3_ [17]. In contrast, the averaged image of A_3_B_3_J, clearly shows extra density in the interior of the central hole but located off center, close to one of the peripheral subunits (Fig. 3b). These observations strongly suggest that FliJ penetrates into the central hole of the A_3_B_3_ complex in a way similar to the D-subunit of V_1_-ATPase [8].

### Rotary motion of FliJ in A_3_B_3_ without uni-directionality

**Figure 4:**
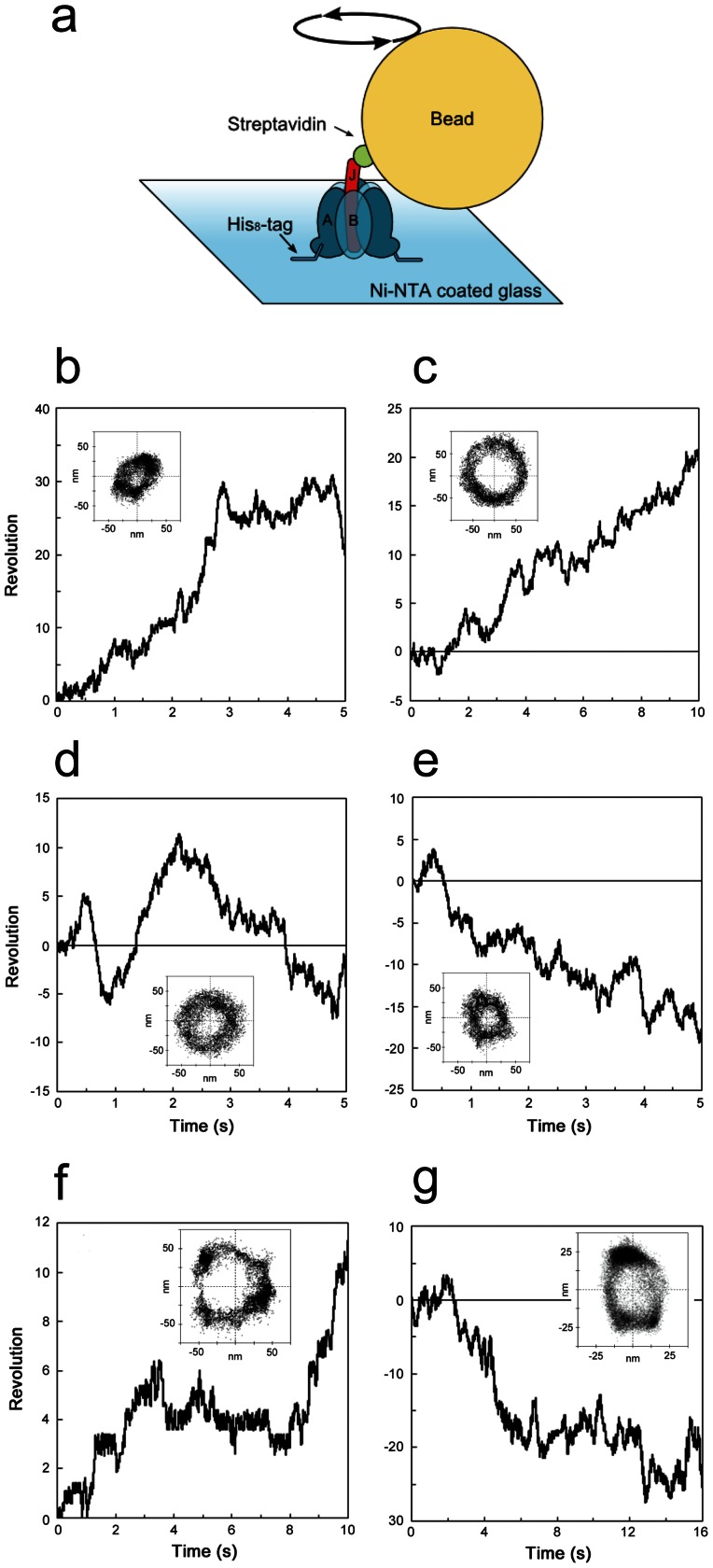
Rotary motion of FliJ in A_3_B_3_. (**a**) Schematic representation of the experimental system. A 40 nm gold particle was attached to FliJ via a streptavidin-biotin linkage. Time courses of the anti-clockwise (**b**, **c**) and clockwise rotation (**d, e**) of beads attached to the shaft of A_3_B_3_J captured at speeds up to 1,000 frames s^−1^ at 2 mM [ATP]. Stepping motions of beads were shown in **f** and **g**. Trajectories of the bead centroid are shown in insets.

We attempted to demonstrate the rotary motion of FliJ against A_3_B_3_ immobilizing A_3_B_3_J on a Ni-NTA-coated glass surface through His_10_-tags introduced at the N terminus of the A subunits, and attaching a 40-nm streptavidin-coated gold colloid (40-nm bead) to the biotin-labelled FliJ (Fig. 4a). Beads were imaged by dark-field microscopy, and beads motions were recorded on a fast-framing CCD camera at speeds up to 1,000 frames s^−1^. We found rotating beads attached to the FliJ in A_3_B_3_. It was confirmed that gold beads observed under the microscope were attached to the A_3_B_3_J complex through FliJ because very few beads were found without A_3_B_3_J. Two to ten beads showing rotary motion were usually found in a single flow cell. However, they did not show apparent uni-directionality. For instance, both rotating beads showing clockwise or anti clockwise rotation (Fig. 4 b–e) were found. Some beads showed stepwise rotary motion (Fig. 4 f,g). The rotary motion of A_3_B_3_J was also observed without ATP in the infusion buffer but the number of beads showing rotary motion apparently decreased; one to three beads showing rotary motion were found in a single flow cell. These results indicate that FliJ in the A_3_B_3_ does not function as a perfect rotor axis. FliJ is only weakly bound to A_3_B_3_, thereby dissociating FliJ from the complex after successive gel filtration chromatography of the A_3_B_3_J complex (Fig. S2b). In agreement with this, the ATPase activity of A_3_B_3_J (7.0 s^−1^), although significantly higher than A_3_B_3_, is much lower than that of V_1_ (65 s^−1^) or A_3_B_3_D (25 s^−1^), reflecting the relative instability of the A_3_B_3_J complex. Therefore, the low binding affinity of the FliJ for A_3_B_3_ is likely to reduce the chance of FliJ rotation being coupled with a conformational change of A_3_B_3_ by ATP hydrolysis.

### Anti-parallel coiled coil structure (FliJ structure) functions as a rotor

**Figure 5:**
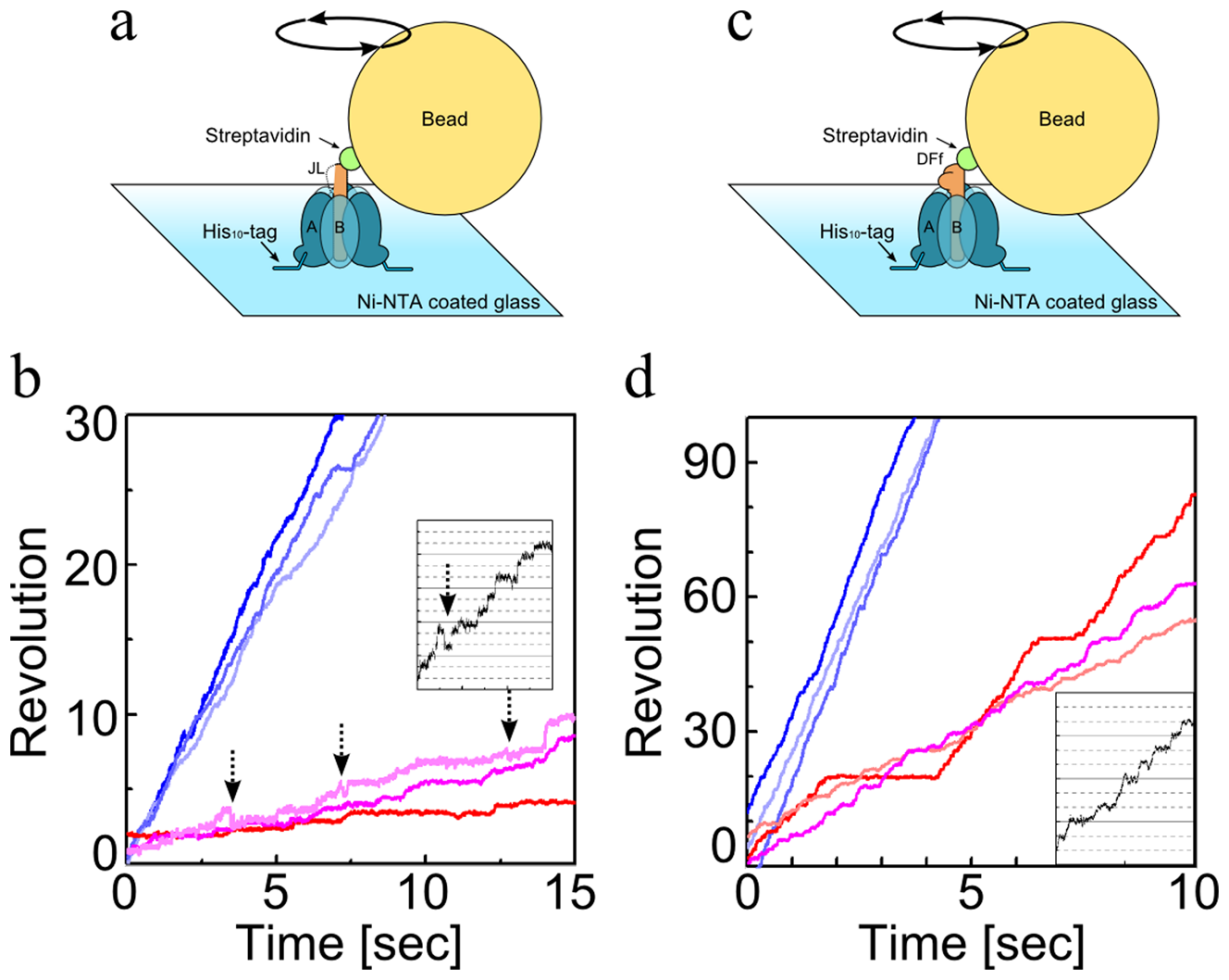
Rotation of A_3_B_3_JL and A_3_B_3_DF_f_. Schematic representation of the experimental system for rotation of A_3_B_3_JL (**a**) and A_3_B_3_DF_f_ (**c**). A magnetic bead was attached to rotor subunit via a streptavidin-biotin linkage. Typical time courses of the rotation of beads attached to the shaft of A_3_B_3_JL (**b**) and A_3_B_3_DF_f_ (**d**) captured at 1000 frames s^−1^ at 10 µM (red lines) and 2 mM [ATP] (blue lines). In insets, the most populated angles for each enzyme are shown by the horizontal lines separated by 120°. In **b**, the movements corresponding to backward step of A_3_B_3_JL are indicated by dashed arrows.

FliJ adopts an anti-parallel coiled coil structure composed of both N- and C-terminal α-helices and is similar to that of V_1_-D or F_1_-γ [13]. If we assume that the rotor subunits of the rotary ATPases and the flagellar type III export apparatus share a common evolutionary origin, then the ancestral rotor subunits adopt an anti-parallel coiled-coil structure, that is, the FliJ structure. In order to demonstrate that the FliJ structure functions as a rotor, we constructed a gene encoding a FliJ structure (FliJ-like protein, termed JL hereafter). The JL gene encodes an anti-parallel coiled coil region of V_1_-D composed of both N- and C-terminal α-helices, without the additional region (52 a.a. length) between the N- and C-terminal helices (Fig. S1a). Then we constructed an expression vector encoding the JL gene along with *atpA* and *atpB* genes (Fig. S3a). The JL protein was co-expressed along with the A and B subunits in *Escherichia coli* and purified the A_3_B_3_JL complex to homogeneity (Fig. 2a). The A_3_B_3_JL was active as an ATPase and the *V*_max_ value of the A_3_B_3_JL is similar to that of A_3_B_3_D (Fig. S3b). Using a direct observation system similar to that described for V_1_ [6], the rotation of the A_3_B_3_JL was able to be visualized (Fig. 5a). We observed rotating beads attached to the A_3_B_3_JL when the flow cell was infused with buffer containing 4 mM or 10 µM ATP (hereafter [ATP]) (Fig. 5b). The results clearly indicate that the JL protein functions as a rotor. At 10 µM [ATP], A_3_B_3_JL showed a clear stepwise rotation, pausing every 120° like F_1_ or V_1_. Interestingly, frequent backward steps were observed in rotation of A_3_B_3_JL but were seldom seen in stepwise rotation of either V_1_ or F_1_, indicating that the extra 53 amino acid residues inserted between the two helices were necessary for continuous rotation without irregular motions (Fig. 5b, inset). Taken together, we propose that an anti-parallel coiled-coil fold unit like FliJ is sufficient to function as a rotor for the ATP-driven rotary motor.

### F_1_-γ like rotor derived from V_1_-D and V_1_-F

As shown in Figure 1, the similar folds of V_1_-F and the globular domain of F_1_-γ prompted us to probe an evolutionary relationship between these two proteins, i.e if could F_1_-γ be derived from two separate proteins as observed for V_1_-D and V_1_-F. To investigate the functional relationship between V_1_-F and globular domain of F_1_-γ, we constructed an expression vector of a mutant V_1_ containing a fusion of the genes coding for subunits D and F. The gene coding for F (*atpF*) was inserted between the two helical regions of the gene coding for D (*atpD*; see Fig. S4a) and the resulting fusion protein was termed DF_f_. The mutant ATPase containing the fused protein (A_3_B_3_DF_f_) was expressed in *E. coli* and purified to homogeneity (Fig. 2a). The A_3_B_3_DF_f_ showed an ATPase activity and exhibited simple Michaelis-Menten kinetics, nearly equal to those of wild type V_1_, which contains separate D and F subunits in the rotor [18]. Using a direct observation system similar to that described for V_1_, rotation was visualized via a bead attached obliquely to the helical region in the DF_f_ (Fig. 5c). We observed rotating beads attached to the A_3_B_3_DF_f_ at 4 mM or 10 µM ATP (Fig. 5d). Stepwise rotation of the A_3_B_3_DF_f_ pausing every 120° was also observed at 10 µM ATP. In this case the frequent backwards steps seen for A_3_B_3_JL were not observed (Figs. 5d, inset). Together, the identical rotation behavior and kinetic parameters of A_3_B_3_DF_f_ confirm that the DF_f_ fully functions as a shaft in the rotary motor. These observations indicate that the V_1_-F when fused to V_1_-D, functions in the rotor in the same way as the globular domain of F_1_-γ and suggest an evolutionary relationship between V_1_-F and V_1_-D and F_1_-γ. In other words, F_1_-γ could be the product of fusion of the genes encoding an ancestral helical protein similar to V_1_-D and an ancestral globular protein similar to V_1_-F.

## Discussion

In this study, we have provided several lines of evidence on the functional similarity of FliJ of the flagellar type III export system to the rotor subunit in rotary ATPases. Analysis of reconstituted A_3_B_3_J revealed that FliJ stabilizes the A_3_B_3_ hexamer by penetrating into A_3_B_3_ and promotes continuous ATPase activity in a manner similar to the V_1_-D subunit. Although FliJ in A_3_B_3_ does not show unidirectional rotation coupling with ATP hydrolysis due to its low binding affinity for A_3_B_3_, The JL protein is sufficient for functioning as a rotor of the ATP-driven rotary motor. Based on very recent high resolution X-ray crystal structure of V_1_ from *E. hirae*, Arai and co-workers have proposed that the interaction of the coiled-coil part of V_1_-D with the A subunit is essential for rotation of V_1_-D against A_3_B_3_ hetero-hexamer [26]. In their V_1_ structure, the globular-loop part of V_1_-DF is rarely in contact with the A_3_B_3_ hexamer. Our results, in which the anti-parallel coiled-coil structure is sufficient as a rotor in rotary ATPases, are consistent with their structural study.

**Figure 6:**
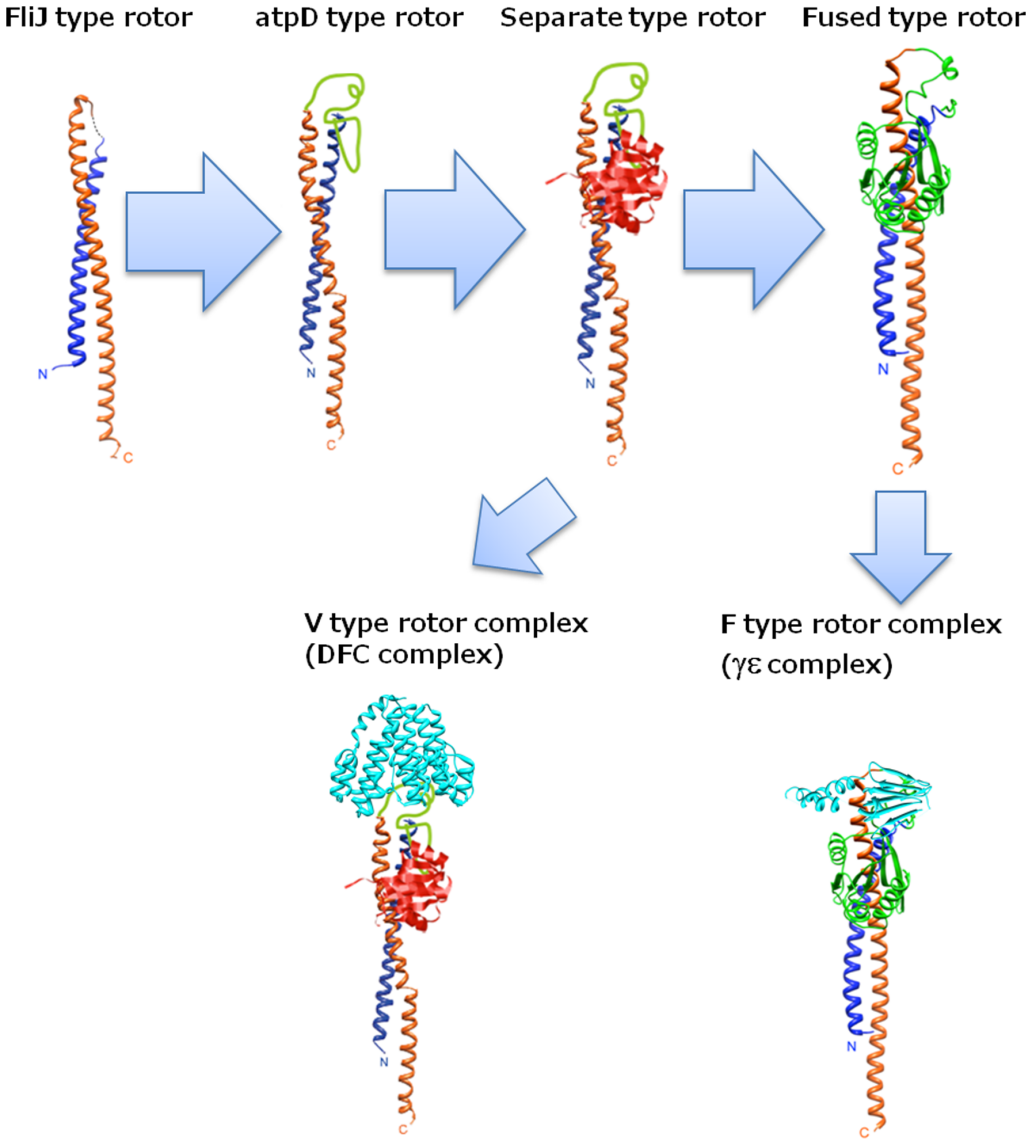
A proposed evolution process for the rotor domains. V_1_-D is evolved from a FliJ like protein by adding loop region. V_1_-D forms a separate rotor shaft together with the globular V_1_-F. F_1_-γ evolved through gene fusion of two genes encoding the ancestral V_1_-D and globular proteins. Assuming that V_o_V_1_ have conserved the ancestral form in the rotor apparatus, C subunit (V_o_-d) has been replaced with F_1_-ε during evolution of F_o_F_1_. Function of each rotor subunit was summarized in Table S1.

Here we propose that V_1_-D, F_1_-γ and FliJ evolved from a common evolutionary origin. The JL protein derived from V_1_-D functioned as a rotor shaft (Figure 5A), strongly suggesting that FliJ maintains features of a prototype rotor. Because the rotor is composed of separate helical and globular subunits in V_o_V_1_, this rotor is an intermediate between the ancestral rotor and the F_1_-γ, which is a single protein containing both helical and globular domains. Therefore, we propose a possible scenario of evolutional process of rotor apparatus in rotary motors (Fig. 6). In this scenario, F_1_-γ evolved from a gene fusion of genes encoding the ancestral V_1_-D and V_1_-F like proteins. However, because there is the sequence and structural diversity between V_1_-F and in the globular domain of F_1_-γ. we cannot exclude the possibility that V_1_-F is not ancestral gene of globular domain of F_1_-γ.

V_1_-F is structurally similar to CheY, a regulatory subunit of the bacterial flagellar motor, which functions to switch the direction of rotation [7]. A phylogenetic tree analysis using the Maximum Likehood (ML) method indicated that V_1_-F is evolutionarily related to CheY rather than to the globular domain of F_1_-γ (Fig. S5). The topology of V_1_-F is also more similar to that of CheY. (Fig. S5). This indicates that V_1_-F and CheY share a common evolutionary origin. In contrast, the sequence and structural diversity between V_1_-F/CheY and in the globular domain of F_1_-γ can be explained. The central rotor apparatus of the two rotary ATPases contain significant structural differences. The V_o_ sector contains the funnel shaped C subunit (V_o_-d subunit), which serves as a socket for the DF rotor in V_1_ [1], [19]. In contrast, F_1_-γ attaches directly onto the F_o_-c ring while the F_1_-ε forms contacts with both the F_1_-γ and F_o_-c ring [1], [3] (see Fig. 1a). It is possible that the differences in contact features between V_1_-F and the globular domain of F_1_-γ have promoted structural and sequence diversity of the rotors during the evolution of the two different ATPases.

In contrast to V_1_-DF and the F_1_-γ, there is little similarity between the other central rotor domain of the V_o_V_1_- and F_o_F_1_ (see Fig. 1a). The F_1_-ε, composed of an N-terminal β sandwich and a short C terminal helix [1], [3] shows neither sequence nor structural similarity to the equivalent V_o_-d (prokaryotic C subunit). Assuming that V_o_V_1_ have conserved the ancestral form in the rotor apparatus, V_o_-d has been replaced with F_1_-ε during evolution of F_o_F_1_.

## Supporting Information

Figure S1 Secondary structure prediction of the D subunit of *T. thermophilus* V-ATPase (**a**), the γ subunit of *E. coli* F_1_. (**b**) and FliJ of *S. enterica* (**c**) using PORTER: http://distill.ucd.ie/porter/. Predicted helical, sheet, and coiled regions are indicated by H, E, and C, respectively. For the γ subunit, both N- and C-terminal helices in the crystal structure (PDB: 1E79) are indicated by black lines. For the D subunit, assigned helices in the crystal structure (PDB: 3A5C) are indicated by black lines. Other regions are disordered in the crystal structure. For the FliJ, both N- and C-terminal helices in the crystal structure (PDB: 3AJW) are indicated by black lines. The site for insertion of the F subunit in the D subunit is indicated with red characters.
(DOC)

Figure S2 (**a**) Analysis of ATPase complexes with gel-permeation chromatography. The mixture of A_3_B_3_ and FliJ was incubated at room temperature for overnight, and then applied onto Superdex-200 equilibrated with 20 mM MOPS (pH 7.0) and 150 mM NaCl. (**b**) SDS-PAGE analysis for A_3_B_3_J after successive gel-permeation chromatography. A_3_B_3_J was further applied onto gel-permeation chromatography, the resultant complex was analyzed by 15% SDS-PAGE (see lane marked 2nd).
(DOC)

Figure S3 (**a**) Construction of the A_3_B_3_JL expression vector. (**b**) ATP hydrolysis activity of A_3_B_3_JL at the indicated [ATP].
(DOC)

Figure S4 (**a**) Construction of A_3_B_3_DF_f_ expression vector. (**b**) ATP hydrolysis acitivity of A_3_B_3_DF_f_ at the indicated [ATP].
(DOC)

Figure S5 Phylogenetic tree of V_1_-F (red circle), the globular domain of F_1_-γ (blue circles), and CheY (green circles). Open circles indicate genes from eukaryotes. Construction of the phylogenetic tree is described in the Methods section.
(DOC)

Table S1 Structure and function of each rotor subunit of Flagellar protein export apparatus, V_o_V_1_, and F_o_F_1_.
(DOC)
