# Supplementary figures and images for: Common Evolutionary Origin for the Rotor Domain of Rotary Atpases and Flagellar Protein Export Apparatus
Source: PLoS One. 2013 May 28;8(5):e64695. doi: 10.1371/journal.pone.0064695 (PMC3665681; doi:10.1371/journal.pone.0064695)

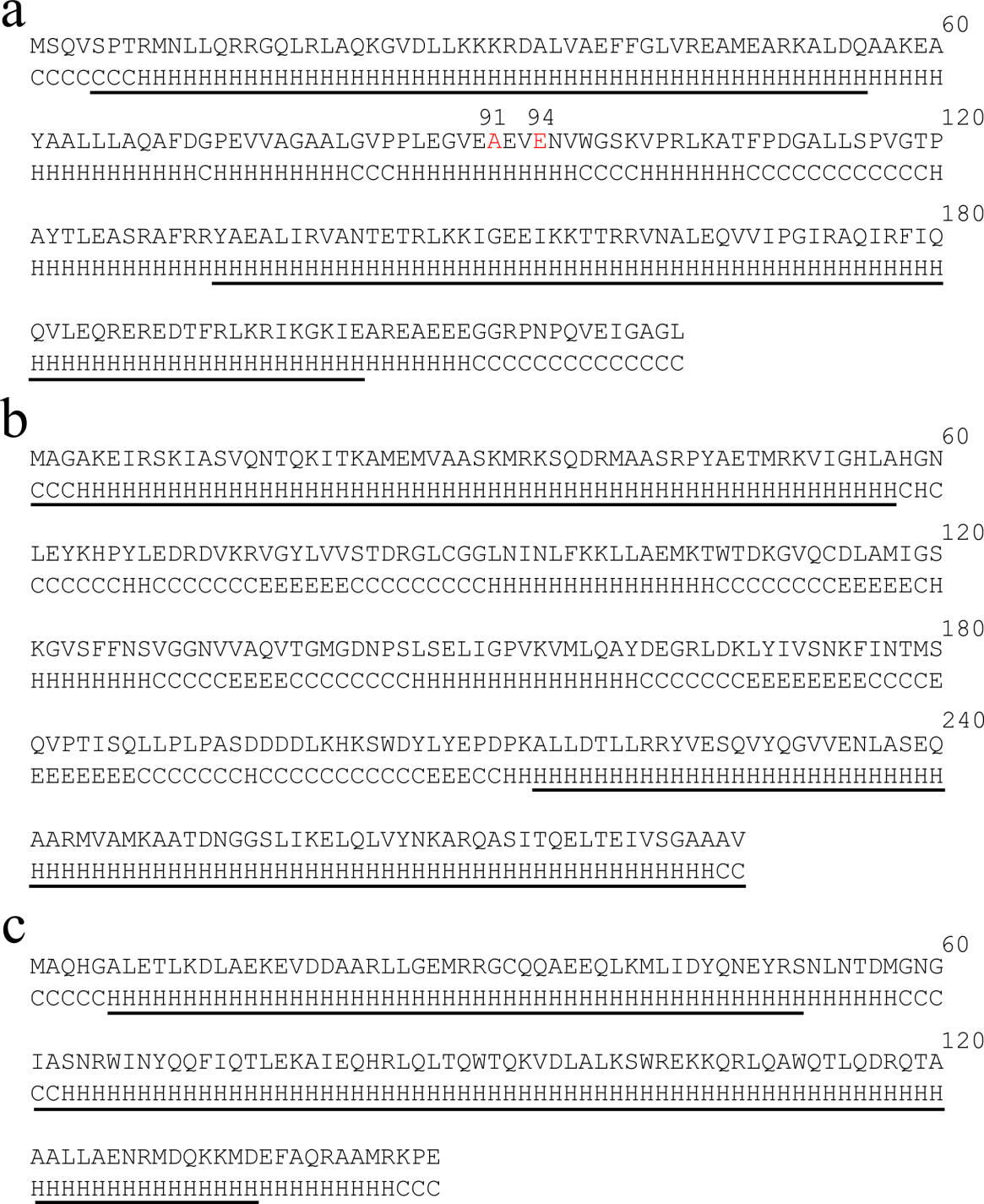


**Figure S1.**

Supplement: Figure S1 — Secondary structure prediction of the D subunit of T. thermophilus V-ATPase (a), the γ subunit of E. coli F1. (b) and FliJ of S. enterica (c) using PORTER: http://distill.ucd.ie/porter/. Predicted helical, sheet, and coiled regions are indicated by H, E, and C, respectively. For the γ subunit, both N- and C-terminal helices in the crystal structure (PDB: 1E79) are indicated by black lines. For the D subunit, assigned helices in the crystal structure (PDB: 3A5C) are indicated by black lines. Other regions are disordered in the crystal structure. For the FliJ, both N- and C-terminal helices in the crystal structure (PDB: 3AJW) are indicated by black lines. The site for insertion of the F subunit in the D subunit is indicated with red characters. (DOC) [file pone.0064695.s001.doc]

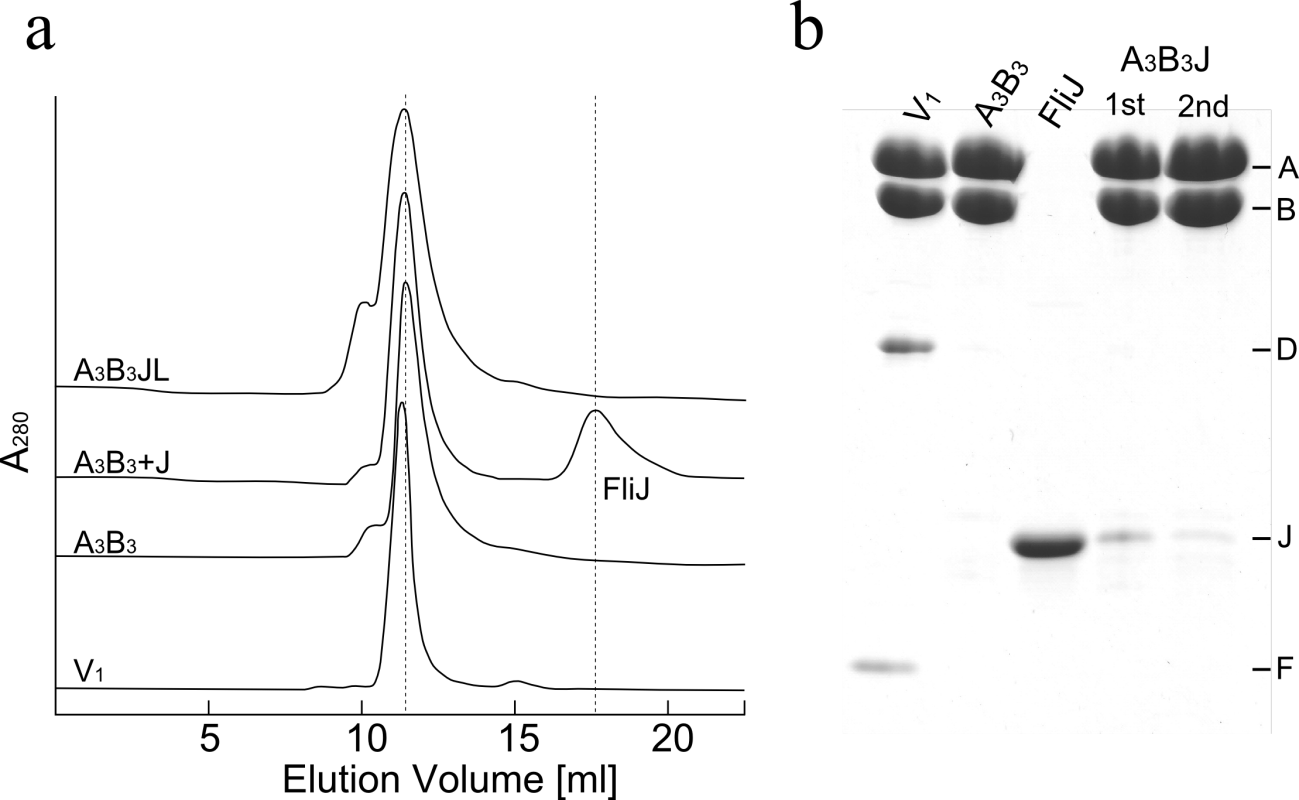


**Figure S2.**

Supplement: Figure S2 — (a) Analysis of ATPase complexes with gel-permeation chromatography. The mixture of A3B3 and FliJ was incubated at room temperature for overnight, and then applied onto Superdex-200 equilibrated with 20 mM MOPS (pH 7.0) and 150 mM NaCl. (b) SDS-PAGE analysis for A3B3J after successive gel-permeation chromatography. A3B3J was further applied onto gel-permeation chromatography, the resultant complex was analyzed by 15% SDS-PAGE (see lane marked 2nd). (DOC) [file pone.0064695.s002.doc]

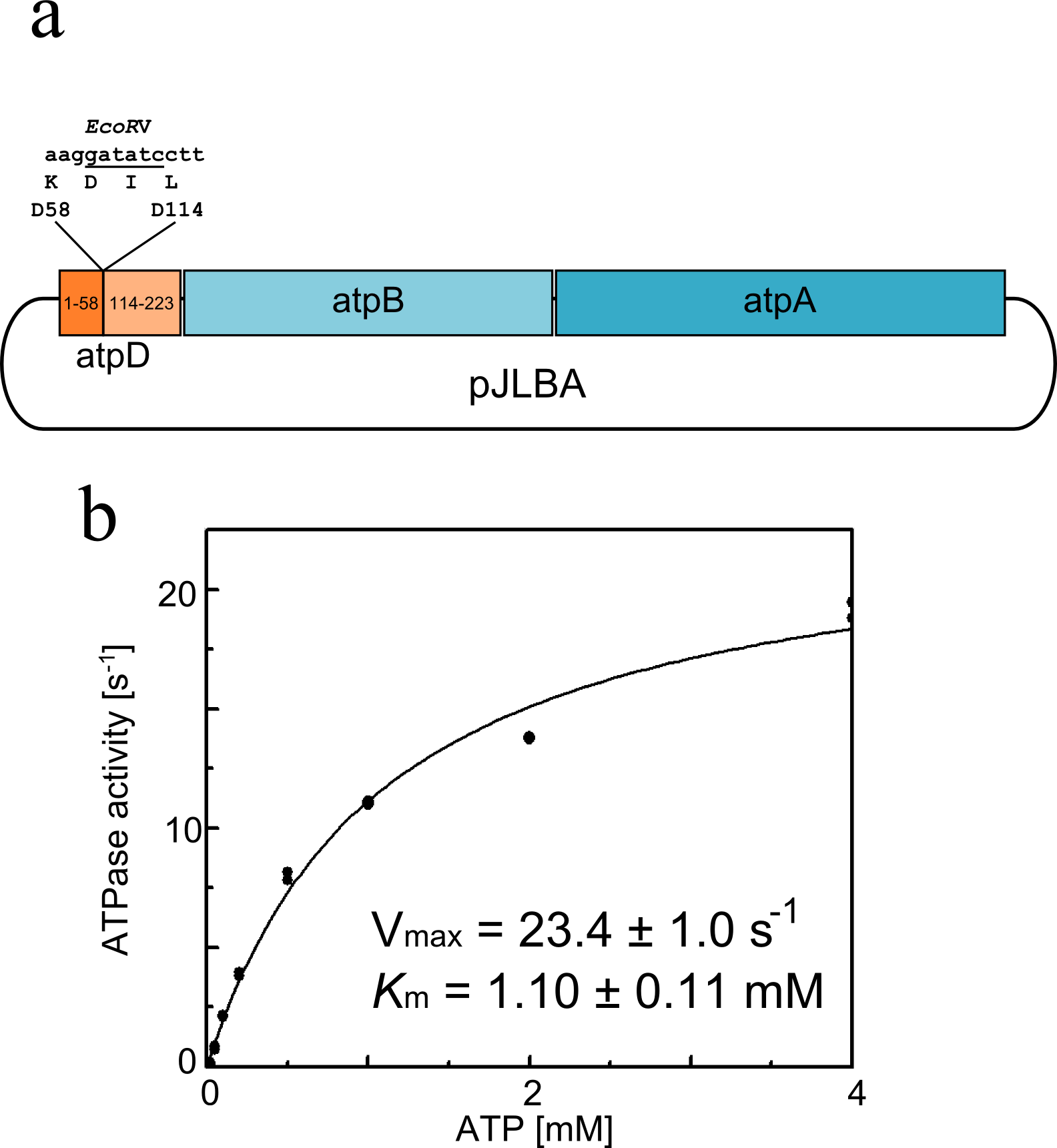


**Figure S3.**

Supplement: Figure S3 — (a) Construction of the A3B3JL expression vector. (b) ATP hydrolysis activity of A3B3JL at the indicated [ATP]. (DOC) [file pone.0064695.s003.doc]

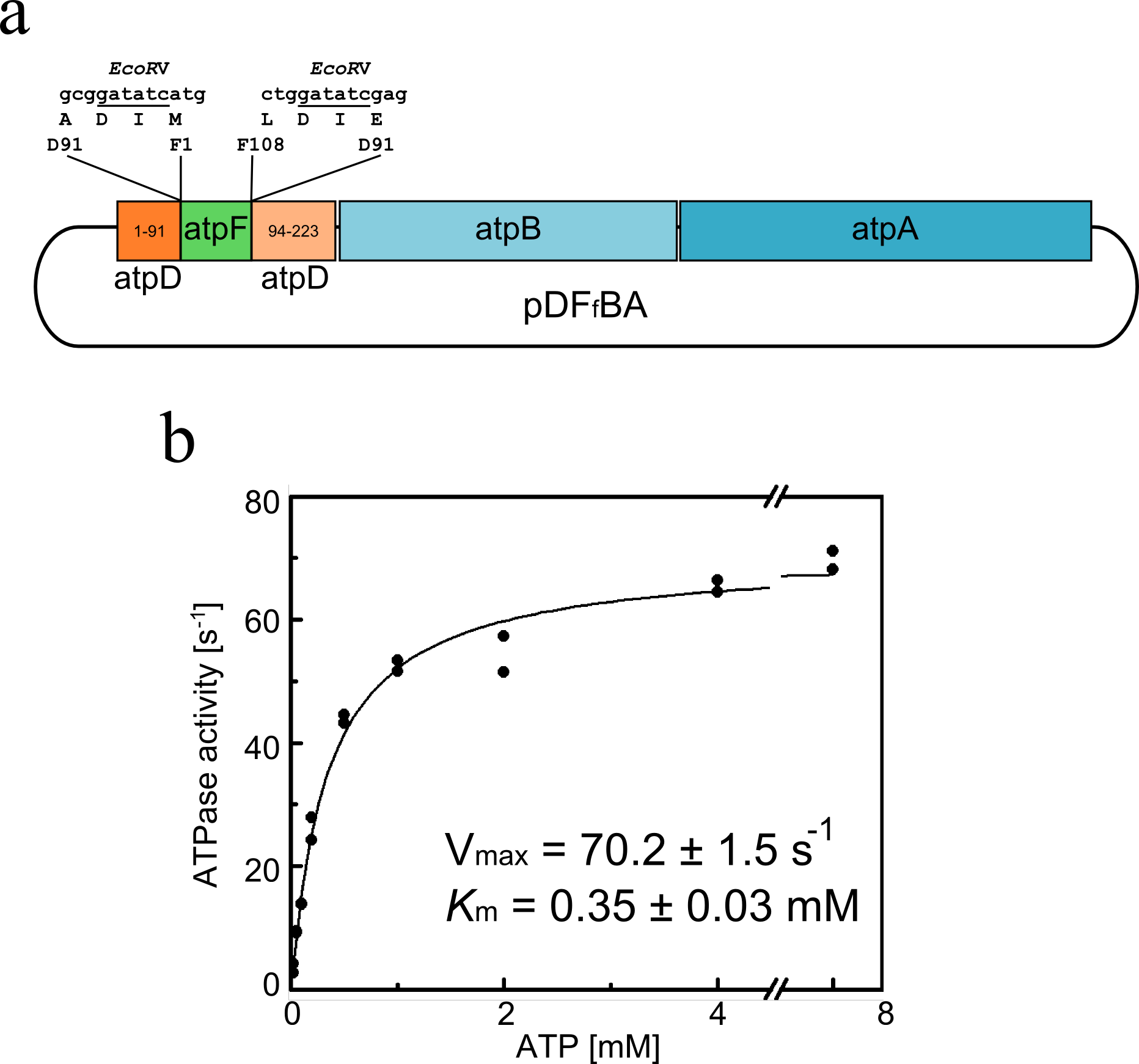


**Figure S4.**

Supplement: Figure S4 — (a) Construction of A3B3DFf expression vector. (b) ATP hydrolysis acitivity of A3B3DFf at the indicated [ATP]. (DOC) [file pone.0064695.s004.doc]

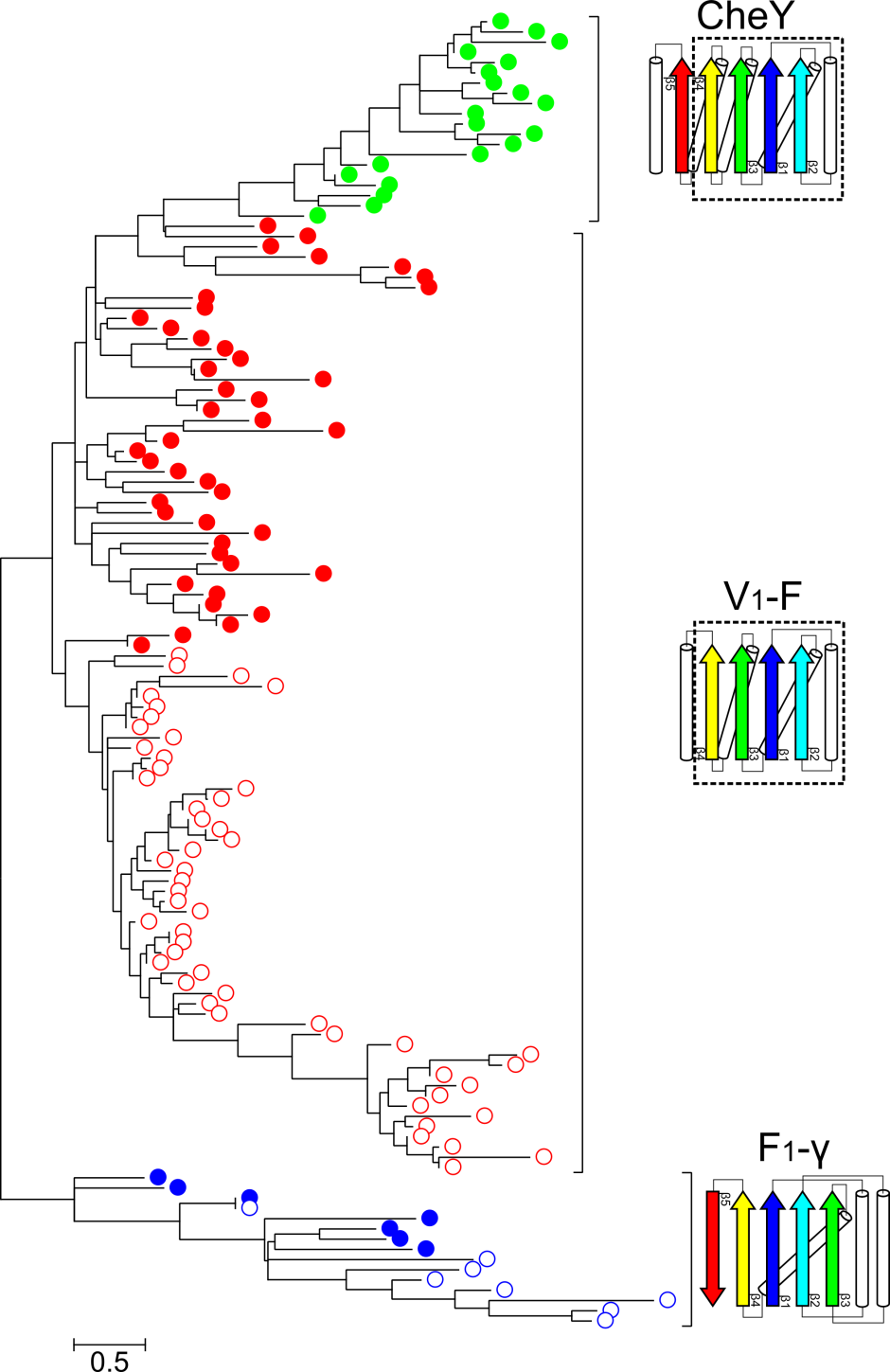


**Figure S5.**

Supplement: Figure S5 — Phylogenetic tree of V1-F (red circle), the globular domain of F1-γ (blue circles), and CheY (green circles). Open circles indicate genes from eukaryotes. Construction of the phylogenetic tree is described in the Methods section. (DOC) [file pone.0064695.s005.doc]
