# Supplementary material for: Common Evolutionary Origin for the Rotor Domain of Rotary Atpases and Flagellar Protein Export Apparatus
Source: PLoS One. 2013 May 28;8(5):e64695. doi: 10.1371/journal.pone.0064695 (PMC3665681; doi:10.1371/journal.pone.0064695)
Supplement: Table S1 — Structure and function of each rotor subunit of Flagellar protein export apparatus, VoV1, and FoF1. (DOC) [file pone.0064695.s006.doc]

|  | Flagellar protein export apparatus | V-ATPases | F_o_F_1_ |
| --- | --- | --- | --- |
| hexamer | FliI_6_ | A_3_B_3_ | α_3_β_3_ |
| Coiled coil domain  (Function) | FliJ  (Stabilization of FliI_6_) | atpD  (Axis) | F_1_−γ subunit  (rotor) |
| Globular domain  (Function) |  | atpF  (torque generation) |
| Regulatory subunit  (Function) |  |  | F_1_-ε subunit  (Regulation) |
| Socket subunit  (Function) |  | C (V_o_-d) subunit  (Association/dissociation of V_1_ part) |  |

Table S1.
